# Retraction: Brain–Computer Interface–Based Communication in the Completely Locked-In State

**DOI:** 10.1371/journal.pbio.3000607

**Published:** 2019-12-16

**Authors:** The PLOS Biology Editors

The editors retract this publication following two investigations into concerns originally brought to the attention to the authors’ institution by a whistleblower. The results of such investigations can be found at the following links:

Eberhard Karls Universität Tübingen
Deutsche Forschungsgemeinschaft

During their investigation, committees at both institutions concluded that the authors of the study are at fault with regard to data collection, handling, and analysis. However, they did not comment on the methodology underlying the findings.

Adhering to the guidelines provided by the Committee on Publication Ethics, the editors retract this manuscript because the authors were found to have engaged in scientific misconduct by Eberhard Karls Universität Tübingen and Deutsche Forschungsgemeinschaft.

The authors have declined to sign this retraction, as they stand by their data, analyses, and conclusions, and state that they intend to take legal proceedings to challenge the findings in the reports, which they claim contain errors.
